# Joint trajectories of adiposity and systemic inflammation and incident diabetes among older adults: a bi-cohort prospective study with mediation analysis

**DOI:** 10.3389/fnut.2026.1867835

**Published:** 2026-08-27

**Authors:** Fubin Teng, Shichao Li, Di Liu

**Affiliations:** Department of Endocrinology and Nephrology, Dongzhimen Hospital, The First Affiliated Hospital of Beijing University of Chinese Medicine, Beijing, China

**Keywords:** adiposity-inflammation trajectories, diabetes, group-based multi-trajectory modeling, mediation analysis, prospective cohort study

## Abstract

**Background:**

Obesity and chronic low-grade inflammation are established risk factors for type 2 diabetes, yet their joint longitudinal evolution and combined contribution to diabetes incidence remain poorly characterized in aging populations.

**Objective:**

To identify joint trajectories of adiposity and systemic inflammation, evaluate their associations with incident diabetes, and quantify the mediating role of glycated hemoglobin (HbA1c) across two independent cohorts.

**Methods:**

This prospective study included 3,733 diabetes-free adults aged ≥50 years from the English Longitudinal Study of Ageing (ELSA, *n* = 1,778) and the Health and Retirement Study (HRS, *n* = 1,955), with approximately 8 years of follow-up. Group-based multi-trajectory modeling identified joint trajectory groups under two strategies: general adiposity (body mass index paired with log-transformed C-reactive protein) and central adiposity (waist-to-height ratio paired with log-transformed C-reactive protein). Associations with incident diabetes were estimated using Cox proportional hazards models pooled via random-effects meta-analysis. Mediation analysis quantified the proportion statistically mediated through mid-wave HbA1c.

**Results:**

Four trajectory groups were identified under each strategy in both cohorts. A graded association was observed: the highest-risk group exhibited substantially elevated diabetes risk under both general adiposity (pooled HR 6.00, 95% CI 3.71–9.70) and central adiposity strategies (pooled HR 5.69, 95% CI 3.37–9.59) after full adjustment. Under the conservative outcome definition, HbA1c mediated 13.7–27.5% of the total effect (25.7–44.8% under the original definition representing an upper bound). Trajectory classification yielded modestly higher C-statistics over baseline cross-classification (ΔC 0.061–0.067, statistically significant in HRS), with significant net reclassification improvement primarily driven by event cases. Results were consistent across strategies and cohorts.

**Conclusion:**

Co-evolving trajectories of adiposity and systemic inflammation exhibit a graded association with incident diabetes in older adults, partially mediated through glycemic deterioration. Trajectory modeling offered modest discriminative gains over single-time-point assessment. These findings support concurrent adiposity-inflammation monitoring for diabetes risk stratification in aging populations.

## Introduction

Diabetes mellitus is among the fastest-growing global health crises, with prevalence projected to exceed 700 million by 2045, imposing substantial cardiovascular, renal, and mortality burdens (1, 2). Obesity, particularly when accompanied by chronic low-grade inflammation, is the principal modifiable risk factor (3), yet the interplay between adiposity and systemic inflammation in driving diabetes risk remains incompletely understood.

Both elevated body mass index (BMI) and C-reactive protein (CRP) independently predict incident diabetes. A meta-analysis of 36 prospective studies (*n* = 125,356) reported a pooled relative risk of 1.77 for the highest versus lowest CRP levels (4). Group-based trajectory modeling has revealed that rapidly increasing BMI trajectories confer the highest diabetes risk (3, 5), while waist-to-height ratio (WHtR), a marker of central adiposity, demonstrates superior predictive value over BMI for cardiometabolic outcomes in certain populations (6). Joint BMI–waist circumference trajectory analyses further indicate that central obesity elevates diabetes risk even among individuals with low BMI trajectories (6). Meanwhile, CRP partially mediates the protective association between healthy dietary patterns and reduced diabetes risk (mediated proportion: 3.7–9.8%) (7), and composite biomarkers such as the CRP-triglyceride-glucose index capture both metabolic and inflammatory risk dimensions (8, 9). Additionally, glycated hemoglobin (HbA1c) trajectories preceding diagnosis vary by obesity status, with obese individuals exhibiting steeper HbA1c increases (10).

Despite these advances, critical gaps persist. Most studies have examined adiposity and inflammation as independent predictors rather than modeling their joint longitudinal evolution (11, 12). Whether general adiposity (BMI) and central adiposity (WHtR) demonstrate concordant or divergent risk patterns when jointly modeled with inflammatory trajectories has not been systematically compared. The mediating role of HbA1c in linking combined adiposity–inflammation trajectories to incident diabetes also remains unexplored, and most existing evidence derives from single-cohort analyses, leaving uncertainty about whether observed associations are generalizable across populations with differing demographic and healthcare characteristics.

To address these gaps, we conducted a bi-cohort prospective study using data from the English Longitudinal Study of Ageing (ELSA) and the Health and Retirement Study (HRS). We employed group-based multi-trajectory modeling to jointly characterize eight-year trajectories of adiposity and inflammation (ln[CRP]) through two parallel strategies—Strategy 1 focusing on general adiposity (BMI) and Strategy 2 on central adiposity (WHtR)—assessed their associations with incident diabetes using Cox proportional hazards models with random-effects meta-analysis, and performed mediation analysis to quantify the mediating contribution of HbA1c. We hypothesized that higher combined adiposity–inflammation trajectories would be associated with progressively greater diabetes incidence, partially mediated through glycemic deterioration, and that these associations would be consistent across both strategies and observed in two independent populations with distinct demographic and healthcare contexts (13–15).

## Materials and methods

### Study design and data sources

This prospective cohort study used harmonized longitudinal data from two population-based aging cohorts: the English Longitudinal Study of Ageing (ELSA) (16) and the Health and Retirement Study (HRS) (17). Both are nationally representative biennial surveys of adults aged ≥50 years. ELSA Waves 2 (2004), 4 (2008), and 6 (2012) and HRS Waves 8 (2006), 10 (2010), and 12 (2014) served as three matched measurement occasions, providing approximately 8 years of follow-up. ELSA and HRS served as two independent cohorts for parallel replication of findings. Ethical approval was obtained from the South Central–Berkshire Research Ethics Committee (17/SC/0588) for ELSA and the University of Michigan Institutional Review Board (HUM00061128) for HRS. This study adhered to the Declaration of Helsinki and followed STROBE guidelines.

### Study population

Eligible participants were aged ≥50 years at baseline. Exclusion criteria were prevalent diabetes at baseline, missing values for all three key biomarkers (body mass index [BMI], waist-to-height ratio [WHtR], and C-reactive protein [CRP]) across waves, and baseline covariate missingness exceeding 30%. The final sample comprised 1,778 ELSA and 1,955 HRS participants.

### Exposure variables and outcome definition

Primary exposures were joint longitudinal trajectories of adiposity and systemic inflammation, modeled via two strategies: Strategy 1 paired BMI (kg/m^2^) with the natural logarithm of CRP (ln[CRP], mg/L) to capture general adiposity–inflammation dynamics; Strategy 2 paired WHtR with ln[CRP] to capture central adiposity–inflammation dynamics. BMI was derived from measured height and weight; WHtR was calculated as waist circumference divided by height; CRP was assayed from venous blood samples using immunoturbidimetric methods in ELSA and latex-enhanced immunoturbidimetric assays applied to dried blood spots in HRS. Standard CRP rather than high-sensitivity CRP was measured at certain waves. This difference in assay methodology between cohorts and across waves represents a potential source of measurement error that may affect trajectory stability and cross-cohort comparability of CRP-based findings. Values below the assay detection limit were set to half the detection limit prior to natural log transformation, which was applied given the right-skewed distribution.

The primary outcome was incident diabetes, defined by self-reported physician diagnosis, initiation of glucose-lowering medication, or glycated hemoglobin (HbA1c) exceeding the diagnostic threshold. Trajectory exposures were measured across three waves (ELSA Waves 2, 4, and 6; HRS Waves 8, 10, and 12), with the mid-wave (ELSA Wave 4; HRS Wave 10) serving as the mediator assessment point. Incident diabetes was ascertained at the final wave (ELSA Wave 6; HRS Wave 12), restricted to participants who were diabetes-free through the mid-wave. This restriction was applied specifically to the mediation analysis to ensure temporal ordering between exposure, mediator, and outcome; participants who developed diabetes before the mid-wave were excluded from the mediation sample but retained in the primary Cox regression analysis. The resulting mediation analytic sample sizes were reported in Supplementary Table 4. Survival time was measured from baseline to diabetes onset or censoring at the final wave. Because trajectory group membership was defined using data spanning the full observation window, including the mid-wave at which the mediator was assessed, the exposure is not strictly temporally prior to the mediator. This inherent feature of trajectory-based mediation analysis should be considered when interpreting mediation estimates.

### Covariates

Covariates were selected based on established associations with adiposity-inflammation pathways and diabetes risk, including age (continuous), gender, education (low/medium/high), marital status, current smoking and drinking status, and baseline comorbidities (hypertension, heart disease, stroke, cancer, chronic lung disease, and arthritis; each binary). Baseline HbA1c was included in fully adjusted models to account for pre-existing glycemic status. Missing covariate data were handled by multiple imputation using chained equations (five imputed datasets), with results pooled using Rubin’s rules. Complete case analysis was performed as a sensitivity analysis.

### Statistical analysis

Continuous variables were summarized as means (standard deviations) or medians (interquartile ranges); categorical variables as frequencies (percentages). Between-cohort differences were tested using Wilcoxon rank-sum and chi-squared tests, with standardized mean differences reported.

Joint trajectory modeling was performed using group-based multi-trajectory modeling (GBMTM) via finite mixture models with Gaussian components (flexmix package). Time functions were specified as quadratic polynomials; linear terms were retained when quadratic models failed to converge. Models with two to six groups were fitted independently in each cohort; the optimal number was determined by Bayesian information criterion, average posterior probability (≥0.80), minimum class proportion (≥5%), and clinical interpretability. Individuals were assigned to the group with the highest posterior probability. Both indicators were standardized to *z*-scores within each cohort prior to modeling. GBMTM accommodates partially observed sequences via maximum likelihood estimation; participants with a minimum of two measurement occasions were retained for trajectory modeling.

Associations between trajectory groups and incident diabetes were estimated using Cox proportional hazards regression with progressive adjustment: Model 1 (age, gender); Model 2 (plus education, marital status, smoking, drinking); Model 3 (plus comorbidities and baseline HbA1c). The proportional hazards assumption was evaluated via Schoenfeld residual tests. Cohort-specific hazard ratios were pooled using DerSimonian–Laird random-effects meta-analysis, with heterogeneity assessed by *I*^2^ and Cochran’s Q. Given that only two cohorts were available (*k* = 2), estimates of between-study variance (*τ*^2^) and *I*^2^ are inherently unstable; accordingly, heterogeneity statistics should be interpreted with caution, and emphasis is placed on the consistency of effect directions and magnitudes across individual cohorts rather than on formal pooled estimates.

Mediation analysis evaluated whether mid-wave HbA1c statistically mediated the trajectory–diabetes association, using the R mediation package. The mediator model was a linear regression of mid-wave HbA1c on trajectory group and covariates. The primary mediation analysis used a conservative diabetes definition (physician diagnosis plus glucose-lowering medication only, excluding the HbA1c diagnostic criterion) with a Cox proportional hazards outcome model to avoid inflation from the overlap between the mediator and the HbA1c-based outcome criterion and to appropriately handle censoring. Average mediation effects (ACME), direct effects, and proportion mediated were estimated via quasi-Bayesian Monte Carlo simulation (1,000 iterations). Identification of mediation effects relies on the sequential ignorability assumption, which requires no unmeasured confounding of the mediator–outcome relationship given observed covariates and no exposure-induced mediator–outcome confounding. Because these assumptions are untestable, sensitivity analyses were conducted by varying the correlation parameter *ρ* between the residuals of the mediator and outcome models to evaluate how robust the mediation estimates were to potential violations, with the crossover *ρ* value at which the average mediation effect equals zero reported. As an upper-bound sensitivity analysis, mediation was also estimated using the original inclusive diabetes definition (incorporating the HbA1c ≥ 6.5% criterion) with a logistic regression outcome model.

Subgroup analyses assessed consistency across demographic and clinical strata; Firth penalized Cox regression was applied to subgroups exhibiting complete or quasi-complete separation, and subgroups with fewer than 10 events were flagged as not estimable. Sensitivity analyses examined robustness under alternative assumptions, including exclusion of early-onset cases (within 2 years), Fine–Gray competing risk models with death as a competing event, complete case analysis, trajectory modeling using raw CRP, and inverse probability of treatment weighting. To evaluate the incremental discriminative value of trajectory classification, the discriminative performance of GBMTM trajectory groups was compared against baseline adiposity–inflammation cross-classification using C-statistics from Cox models, with the significance of differences assessed by the method of DeLong et al. This comparison should be interpreted with the caveat that the trajectory model incorporates three waves of measurement whereas the baseline comparator uses a single time point, so any improvement may partially reflect the additional data rather than the trajectory shape per se. Model fit was further compared using the Akaike information criterion and Bayesian information criterion. Continuous net reclassification improvement with bootstrap 95% confidence intervals quantified the reclassification accuracy gained by trajectory classification. All tests were two-sided (*p* < 0.05). Analyses were conducted using R version 4.3.0 with flexmix (2.3–19), survival (3.5–7), meta (6.5–0), mediation (4.5.0), mice (3.16.0), coxphf (1.13.4), nricens (1.6), tableone (0.13.2), and forestploter (1.1.1).

## Results

### Study population and baseline characteristics

**Figure 1:**
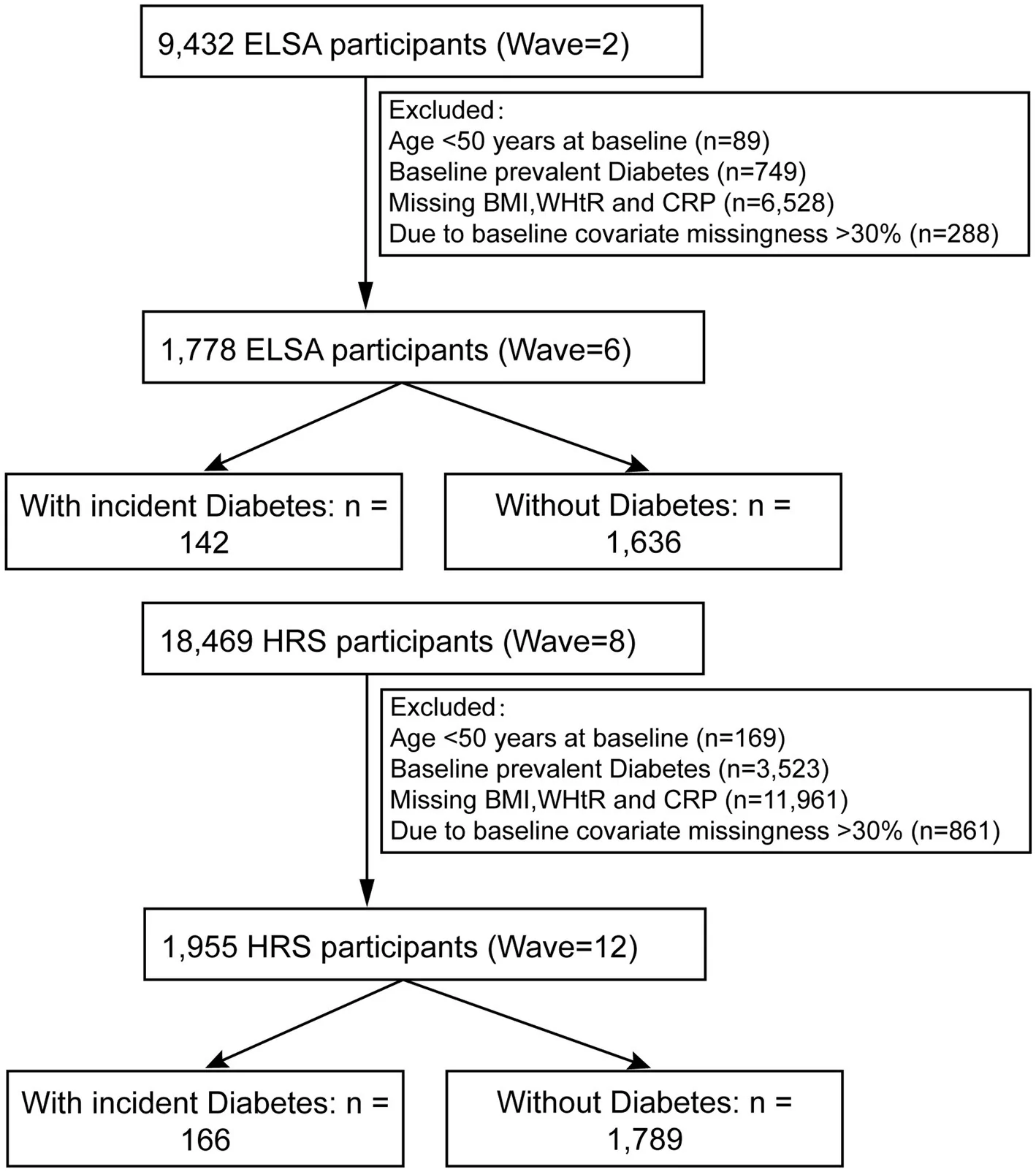
Flow diagram of participant selection. This figure illustrates the sequential application of inclusion and exclusion criteria in ELSA (starting from Wave 2) and HRS (starting from Wave 8), including exclusions for age below 50 years, prevalent diabetes at baseline, missing key biomarker data (BMI, WHtR, and CRP), and baseline covariate missingness exceeding 30%. Final analytic samples and numbers of incident diabetes cases and diabetes-free participants are shown for each cohort.

Of 9,432 ELSA and 18,469 HRS participants initially identified, 7,654 and 16,514 were excluded, respectively, due to age ineligibility, prevalent diabetes, missing key biomarkers, or excessive covariate missingness, yielding a final analytic sample of 3,733 individuals (ELSA: 1,778; HRS: 1,955) (Figure 1). A total of 142 incident diabetes cases were identified in ELSA and 166 in HRS during follow-up. Comparison of baseline characteristics between included and excluded participants revealed that included individuals were more likely to be married, non-smoking, and free of cardiovascular comorbidities, although incident diabetes rates were comparable between groups (both approximately 8%, all SMD < 0.12) (Supplementary Table 1). HRS participants were older (66.11 ± 9.37 vs. 62.43 ± 6.93 years, *p* < 0.001), had higher WHtR (0.60 ± 0.08 vs. 0.57 ± 0.07, *p* < 0.001), and greater prevalence of hypertension (47.6% vs. 31.1%), heart disease (16.0% vs. 2.5%), and arthritis (52.1% vs. 30.6%) (all *p* < 0.001), while BMI was comparable (SMD = 0.055). Current drinking was substantially more prevalent in ELSA (93.6% vs. 56.8%, *p* < 0.001). Detailed baseline characteristics are presented in Table 1.

**Table 1:** Baseline characteristics of study participants by database.

Characteristics	Level	Overall (*n* = 3,733)	ELSA (*n* = 1778)	HRS (*n* = 1955)	*p*	SMD
Age, years		64.36 (8.50)	62.43 (6.93)	66.11 (9.37)	<0.001	0.298
Body mass index, kg/m^2^		27.82 (4.94)	27.61 (4.54)	28.02 (5.27)	0.041	0.055
Waist-to-height ratio		0.58 (0.08)	0.57 (0.07)	0.60 (0.08)	<0.001	0.294
C-reactive protein, mg/L		1.80 [0.82, 3.91]	1.70 [0.80, 3.60]	1.89 [0.87, 4.27]	0.016	0.078
ln(CRP), mg/L		0.61 (1.14)	0.56 (1.09)	0.66 (1.20)	0.027	0.059
Glycated hemoglobin (HbA1c), %		5.51 (0.50)	5.44 (0.45)	5.58 (0.54)	<0.001	0.177
Gender	Female	2021 (54.1)	988 (55.6)	1,033 (52.8)	0.247	0.037
	Male	1712 (45.9)	790 (44.4)	922 (47.2)		
Education level	Low	783 (21.0)	507 (28.5)	276 (14.1)	<0.001	0.246
	Medium	2,126 (57.0)	950 (53.4)	1,176 (60.2)		
	High	824 (22.1)	321 (18.1)	503 (25.7)		
Marital status	Divorced	574 (15.4)	207 (11.6)	367 (18.8)	<0.001	0.220
	Married	2,492 (66.8)	1,327 (74.6)	1,165 (59.6)		
	Single	183 (4.9)	78 (4.4)	105 (5.4)		
	Widowed	484 (13.0)	166 (9.3)	318 (16.3)		
Current smoking	No	3,275 (87.7)	1,578 (88.8)	1,697 (86.8)	0.194	0.040
	Yes	458 (12.3)	200 (11.2)	258 (13.2)		
Current drinking	No	957 (25.6)	113 (6.4)	844 (43.2)	<0.001	0.621
	Yes	2,776 (74.4)	1,665 (93.6)	1,111 (56.8)		
Hypertension	No	2,250 (60.3)	1,225 (68.9)	1,025 (52.4)	<0.001	0.227
	Yes	1,483 (39.7)	553 (31.1)	930 (47.6)		
Heart disease	No	3,377 (90.5)	1734 (97.5)	1,643 (84.0)	<0.001	0.324
	Yes	356 (9.5)	44 (2.5)	312 (16.0)		
Stroke	No	3,615 (96.8)	1748 (98.3)	1867 (95.5)	<0.001	0.110
	Yes	118 (3.2)	30 (1.7)	88 (4.5)		
Cancer	No	3,413 (91.4)	1,682 (94.6)	1731 (88.5)	<0.001	0.147
	Yes	320 (8.6)	96 (5.4)	224 (11.5)		
Chronic lung disease	No	3,539 (94.8)	1717 (96.6)	1822 (93.2)	<0.001	0.103
	Yes	194 (5.2)	61 (3.4)	133 (6.8)		
Arthritis	No	2,170 (58.1)	1,234 (69.4)	936 (47.9)	<0.001	0.297
	Yes	1,563 (41.9)	544 (30.6)	1,019 (52.1)		

Data are presented as mean (SD), median [IQR], or *n* (%). BMI, body mass index; WHtR, waist-to-height ratio; CRP, C-reactive protein; HbA1c, glycated hemoglobin. CRP is presented as median [IQR] due to skewed distribution; ln(CRP) = natural log-transformed CRP. *p*-values from Wilcoxon rank-sum test (continuous) or Chi-squared test (categorical) between the two databases. SMD, standardized mean difference. ELSA, english longitudinal study of ageing; HRS, health and retirement study. Baseline wave: ELSA Wave 2, HRS wave 8. Strategy 1: BMI + ln(CRP) joint trajectory; Strategy 2: WHtR + ln(CRP) joint trajectory. Data are presented as mean (standard deviation) for normally distributed variables, median [interquartile range] for CRP due to skewed distribution, or *n* (%) for categorical variables. Between-cohort comparisons were performed using Wilcoxon rank-sum tests for continuous variables and chi-squared tests for categorical variables. Standardized mean differences are reported. BMI, body mass index; WHtR, waist-to-height ratio; CRP, C-reactive protein; ln(CRP), natural log-transformed CRP; HbA1c, glycated hemoglobin. Baseline defined as ELSA Wave 2 (2004) and HRS Wave 8 (2006). Baseline characteristics of study participants by database.

### Temporal trends and correlations of adiposity and inflammatory biomarkers

**Figure 2:**
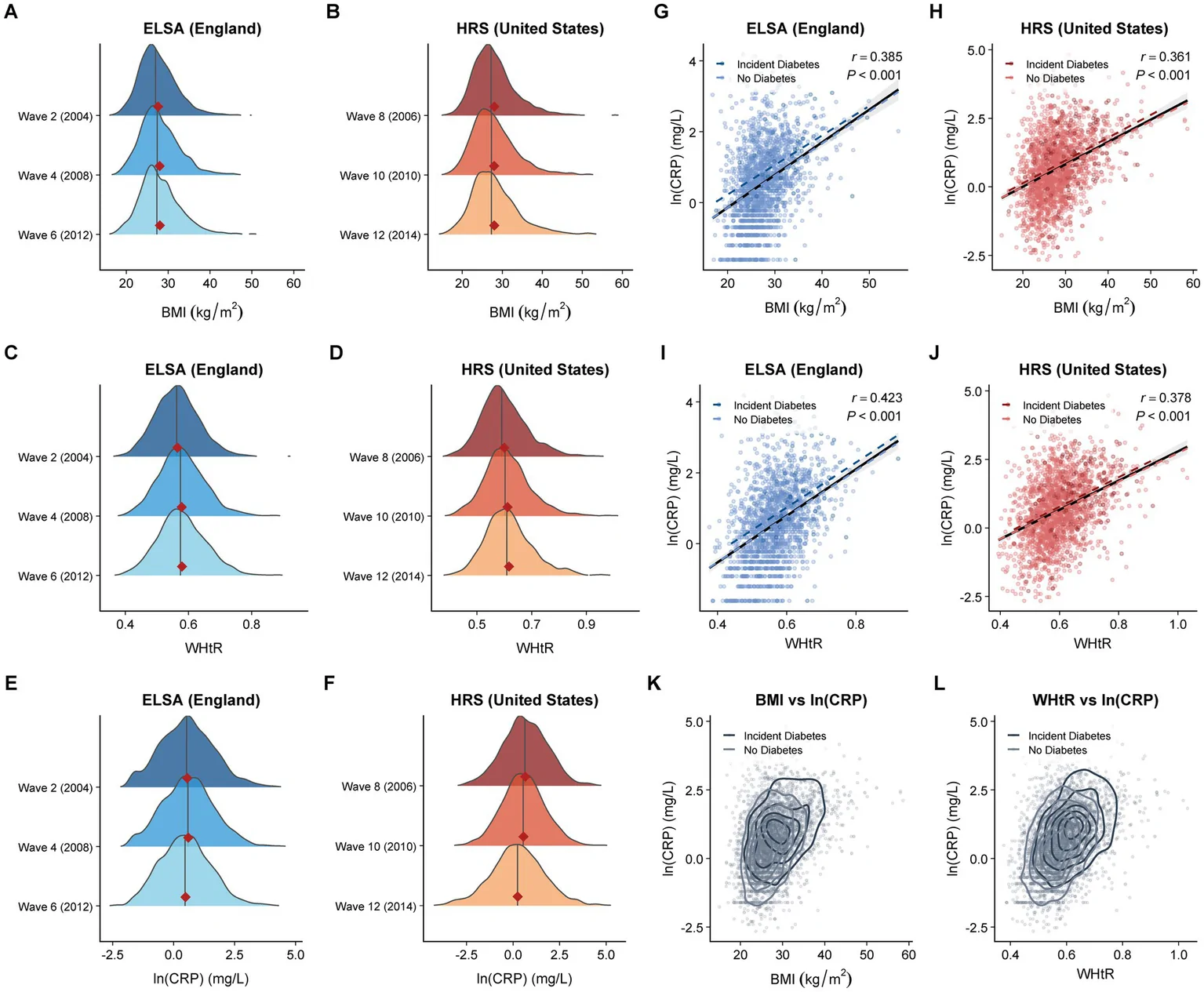
Distribution of adiposity and inflammatory biomarkers across study waves and their baseline correlations. Panels **(A–F)** display wave-specific density distributions of BMI, WHtR, and ln(CRP) in ELSA (Waves 2, 4, and 6) and HRS (Waves 8, 10, and 12), with vertical lines indicating median values and diamond markers indicating means. Panels **(G–J)** present baseline scatter plots of BMI versus ln(CRP) and WHtR versus ln(CRP), stratified by diabetes outcome, with Pearson correlation coefficients and linear regression fit lines. Panels **(K,L)** show two-dimensional kernel density contour plots of BMI versus ln(CRP) and WHtR versus ln(CRP) for the pooled baseline sample, stratified by incident diabetes status. BMI, body mass index; WHtR, waist-to-height ratio; CRP, C-reactive protein; ln(CRP), natural log-transformed CRP.

The temporal patterns of adiposity and inflammation across waves were first examined. BMI remained stable in both cohorts (range: 27.61–28.02 kg/m^2^). WHtR showed a modest upward trend (ELSA: 0.57 to 0.58; HRS: 0.60 to 0.62). Mean ln(CRP) was relatively stable in ELSA (0.49–0.60) but declined in HRS from 0.66 to 0.25 (Figures 2A–F). At baseline, participants who subsequently developed diabetes had higher BMI, WHtR, and ln(CRP) than those who remained diabetes-free in both cohorts (Figures 2G–J). Pearson correlations between adiposity indicators and ln(CRP) were moderate and consistent: BMI versus ln(CRP) (ELSA: *r* = 0.385; HRS: *r* = 0.361) and WHtR versus ln(CRP) (ELSA: *r* = 0.423; HRS: *r* = 0.378) (all *p* < 0.001). Two-dimensional density distributions further illustrated joint clustering patterns by diabetes status (Figures 2K,L).

### Identification of joint trajectory groups

**Figure 3:**
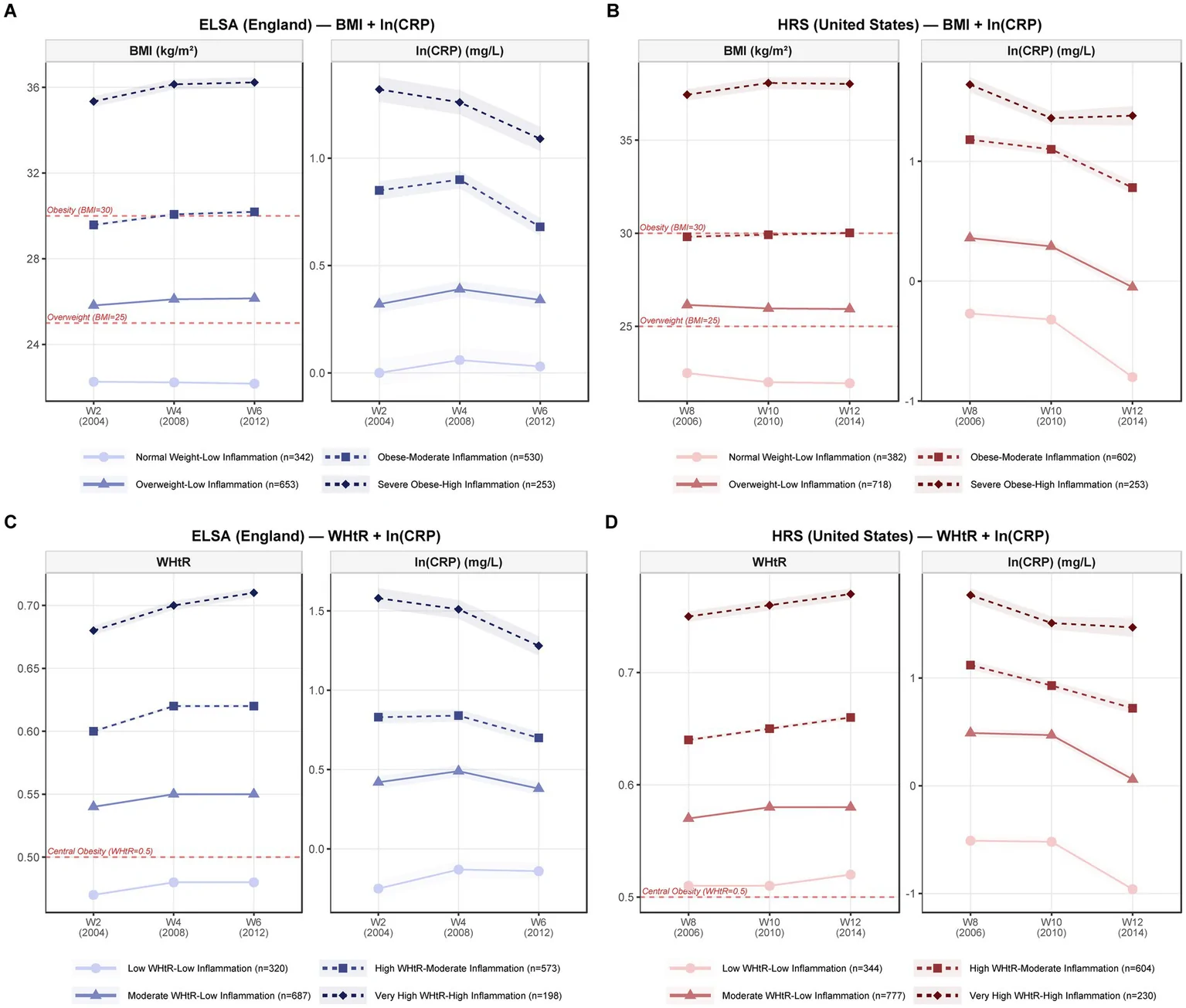
Joint trajectory groups identified by group-based multi-trajectory modeling. **(A)** Shows ELSA Strategy 1 (BMI + ln[CRP]) trajectories; **(B)** Shows HRS Strategy 1 trajectories; **(C)** Shows ELSA Strategy 2 (WHtR + ln[CRP]) trajectories; **(D)** Shows HRS Strategy 2 trajectories. Each panel displays mean values with standard error ribbons across three measurement waves for four trajectory groups, with separate facets for each indicator. Dashed horizontal reference lines indicate clinical thresholds (BMI = 25 and 30 kg/m^2^ for overweight and obesity; WHtR = 0.5 for central obesity). Group sample sizes are indicated in the legend.

Joint trajectory groups were then identified to characterize co-evolving patterns of adiposity and inflammation. GBMTM with four groups was selected as optimal for both strategies in each cohort. Under the general adiposity strategy (BMI + ln[CRP]), four groups were identified in ELSA: Normal Weight–Low Inflammation (*n* = 342, 19.2%), Overweight–Low Inflammation (*n* = 653, 36.7%), Obese–Moderate Inflammation (*n* = 530, 29.8%), and Severe Obese–High Inflammation (*n* = 253, 14.2%) (Figure 3A). HRS showed consistent distributions: 19.5, 36.7, 30.8, and 12.9%, respectively (Figure 3B). BMI trajectories remained stable within each group, while ln(CRP) levels tracked proportionally with adiposity.

Under the central adiposity strategy (WHtR + ln[CRP]), four analogous groups emerged in ELSA: Low WHtR–Low Inflammation (18.0%), Moderate WHtR–Low Inflammation (38.6%), High WHtR–Moderate Inflammation (32.2%), and Very High WHtR–High Inflammation (11.1%) (Figure 3C). HRS yielded comparable proportions: 17.6, 39.7, 30.9, and 11.8% (Figure 3D). Both strategies demonstrated concordant trajectory structures across cohorts, with progressively higher adiposity coupled with escalating inflammatory burden.

### Association between joint trajectories and incident diabetes

**Figure 4:**
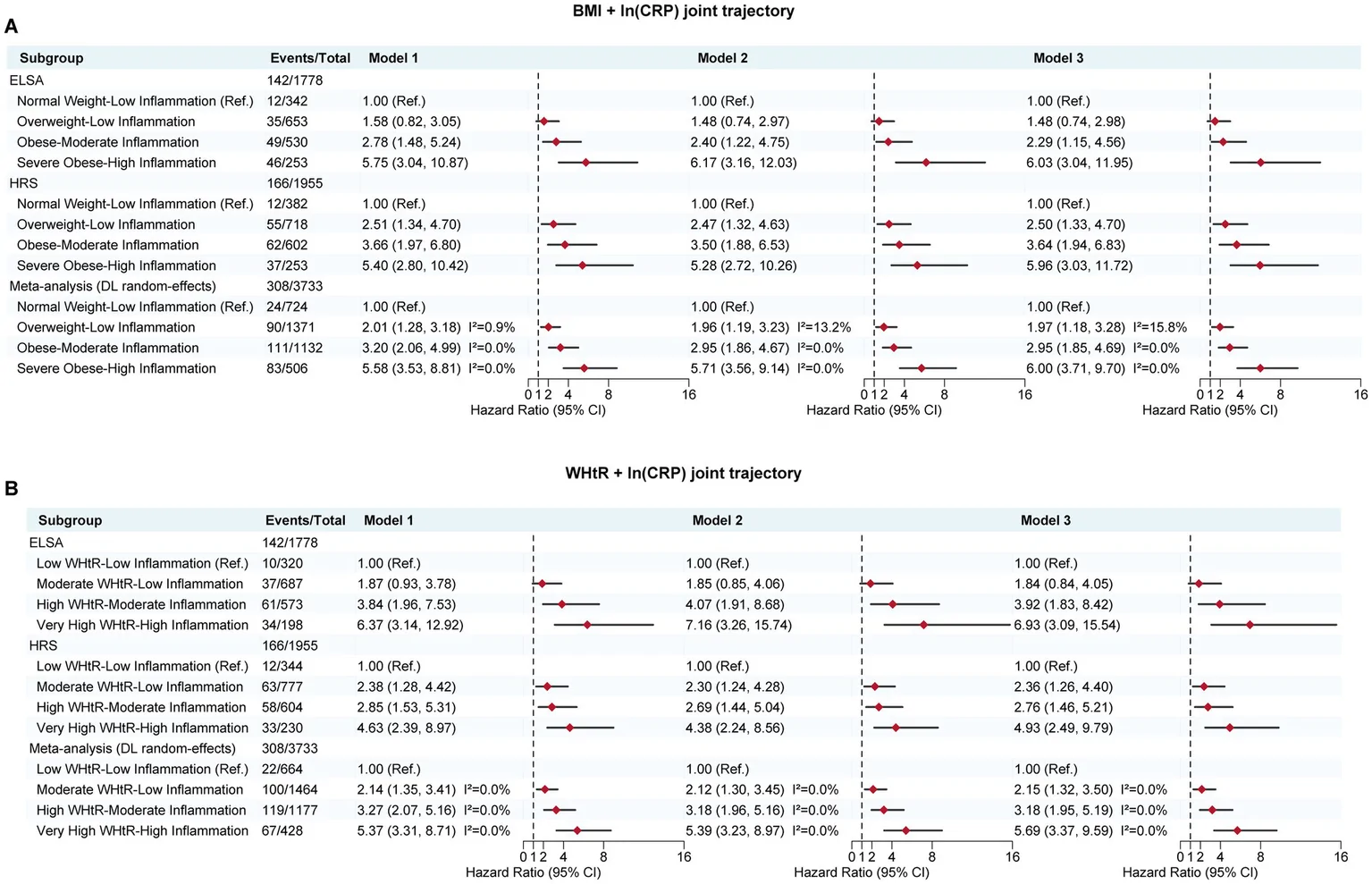
Forest plots of hazard ratios from Cox proportional hazards models and random-effects meta-analysis for incident diabetes by trajectory group. Panel **(A)** presents results for Strategy 1 (BMI + ln[CRP] joint trajectory) and Panel **(B)** for Strategy 2 (WHtR + ln[CRP] joint trajectory). Three progressively adjusted models are displayed: Model 1 (age and gender); Model 2 (Model 1 plus education, marital status, smoking, and drinking); Model 3 (Model 2 plus hypertension, heart disease, stroke, cancer, chronic lung disease, arthritis, and baseline HbA_1c_). Cohort-specific estimates from ELSA and HRS are pooled using DerSimonian-Laird random-effects meta-analysis, with *I*^2^ statistics for heterogeneity. The dashed vertical line indicates the null value (*HR* = 1.00). HR, hazard ratio; CI, confidence interval; DL, DerSimonian-Laird.

The association between trajectory group membership and diabetes risk was subsequently evaluated. The proportional hazards assumption was satisfied for all models (global Schoenfeld *p* > 0.05). In the fully adjusted Model 3 under the general adiposity strategy, compared with the Normal Weight–Low Inflammation reference, the Obese–Moderate Inflammation group showed significantly elevated risk in both ELSA (HR 2.29, 95% CI 1.15–4.56, *p* = 0.018) and HRS (HR 3.64, 95% CI 1.94–6.83, *p* < 0.001). The Severe Obese–High Inflammation group exhibited the highest risk (ELSA: HR 6.03, 95% CI 3.04–11.95; HRS: HR 5.96, 95% CI 3.03–11.72; both *p* < 0.001). The Overweight–Low Inflammation group reached significance in HRS (HR 2.50, 95% CI 1.33–4.70, *p* = 0.004) but not in ELSA (HR 1.48, 95% CI 0.74–2.98, *p* = 0.267) (Figure 4A).

The central adiposity strategy yielded a similar graded association pattern. The Very High WHtR–High Inflammation group had the highest risk (ELSA: HR 6.93, 95% CI 3.09–15.54; HRS: HR 4.93, 95% CI 2.49–9.79; both *p* < 0.001), followed by the High WHtR–Moderate Inflammation group (ELSA: HR 3.92, 95% CI 1.83–8.42; HRS: HR 2.76, 95% CI 1.46–5.21) (Figure 4B). Hazard ratio magnitudes were broadly comparable between strategies at corresponding risk tiers and remained stable across progressive adjustment models, indicating that both general and central adiposity–inflammation trajectories conferred similar graded diabetes risk, largely independent of sociodemographic, behavioral, and comorbidity confounders. Pooled meta-analytic estimates are presented in Figure 4.

### Subgroup and sensitivity analyses

The robustness of primary findings was further assessed through subgroup and sensitivity analyses. Subgroup analyses stratified by age, gender, education, smoking, drinking, and baseline comorbidities demonstrated that the graded trajectory–diabetes association was broadly consistent across strata. Interaction tests were largely non-significant, with isolated exceptions: arthritis in ELSA under the general adiposity strategy (*p* = 0.021), smoking in ELSA under the central adiposity strategy (*p* = 0.040), and age in HRS under the central adiposity strategy (*p* = 0.035). Subgroups with fewer than 10 events were flagged as not estimable, and Firth penalized Cox regression was applied to sparse-data subgroups exhibiting complete or quasi-complete separation; however, confidence intervals remained non-estimable in these subgroups and are reported as such (Supplementary Table 2). As six pairwise comparisons were performed for each strategy, isolated significant interactions should be interpreted with caution in the context of multiplicity.

Sensitivity analyses confirmed result stability across multiple alternative approaches. Exclusion of early-onset cases within 2 years yielded comparable hazard ratios for the highest-risk group (Severe Obese–High Inflammation: ELSA HR 5.62, 95% CI 2.76–11.43; HRS HR 5.58, 95% CI 2.77–11.24). Fine–Gray competing risk models accounting for death as a competing event (ELSA HR 5.28, 95% CI 2.62–10.63; HRS HR 5.21, 95% CI 2.61–10.40), complete case analysis (ELSA HR 6.18, 95% CI 3.02–12.64; HRS HR 6.12, 95% CI 3.01–12.44), trajectory modeling using raw CRP (ELSA HR 5.74, 95% CI 2.85–11.57; HRS HR 5.68, 95% CI 2.84–11.35), and inverse probability of treatment weighting (ELSA HR 5.42, 95% CI 2.65–11.09; HRS HR 5.38, 95% CI 2.66–10.89) all produced materially unchanged results. Consistent patterns were observed under the central adiposity strategy. A summary of hazard ratios across all sensitivity scenarios under both strategies is presented in Table 2, with full details in Supplementary Table 3.

**Table 2:** Summary of sensitivity analyses for the association between adiposity-inflammation trajectories and incident diabetes (Model 3 HR, 95% CI).

Sensitivity analysis	Database	Strategy	Group 2 vs Ref	Group 3 vs Ref	Group 4 vs Ref
Primary analysis	ELSA	Strategy 1	1.48 (0.74, 2.98)	2.29 (1.15, 4.56)	6.03 (3.04, 11.95)
	HRS	Strategy 1	2.50 (1.33, 4.70)	3.64 (1.94, 6.83)	5.96 (3.03, 11.72)
	ELSA	Strategy 2	1.84 (0.84, 4.05)	3.92 (1.83, 8.42)	6.93 (3.09, 15.54)
	HRS	Strategy 2	2.36 (1.26, 4.40)	2.76 (1.46, 5.21)	4.93 (2.49, 9.79)
Excluding DM ≤ 2 years	ELSA	Strategy 1	1.38 (0.67, 2.83)	2.14 (1.05, 4.36)	5.62 (2.76, 11.43)
	HRS	Strategy 1	2.34 (1.22, 4.49)	3.41 (1.78, 6.53)	5.58 (2.77, 11.24)
	ELSA	Strategy 2	1.72 (0.76, 3.89)	3.65 (1.66, 8.03)	6.45 (2.80, 14.86)
	HRS	Strategy 2	2.21 (1.16, 4.22)	2.58 (1.34, 4.97)	4.61 (2.27, 9.37)
Fine-Gray competing risk	ELSA	Strategy 1	1.35 (0.66, 2.76)	2.06 (1.02, 4.16)	5.28 (2.62, 10.63)
	HRS	Strategy 1	2.26 (1.18, 4.33)	3.24 (1.70, 6.17)	5.21 (2.61, 10.40)
	ELSA	Strategy 2	1.65 (0.74, 3.68)	3.46 (1.58, 7.57)	6.02 (2.64, 13.72)
	HRS	Strategy 2	2.12 (1.12, 4.01)	2.48 (1.30, 4.73)	4.32 (2.15, 8.68)
Complete case	ELSA	Strategy 1	1.52 (0.73, 3.16)	2.35 (1.14, 4.84)	6.18 (3.02, 12.64)
	HRS	Strategy 1	2.58 (1.33, 5.01)	3.72 (1.92, 7.21)	6.12 (3.01, 12.44)
	ELSA	Strategy 2	1.89 (0.83, 4.30)	4.02 (1.81, 8.93)	7.14 (3.06, 16.65)
	HRS	Strategy 2	2.42 (1.25, 4.68)	2.84 (1.45, 5.56)	5.08 (2.48, 10.41)
Raw CRP	ELSA	Strategy 1	1.42 (0.70, 2.88)	2.18 (1.08, 4.40)	5.74 (2.85, 11.57)
	HRS	Strategy 1	2.38 (1.25, 4.53)	3.48 (1.83, 6.62)	5.68 (2.84, 11.35)
	ELSA	Strategy 2	1.76 (0.79, 3.93)	3.72 (1.71, 8.09)	6.58 (2.88, 15.03)
	HRS	Strategy 2	2.24 (1.18, 4.25)	2.62 (1.37, 5.01)	4.68 (2.33, 9.40)
IPTW	ELSA	Strategy 1	1.36 (0.65, 2.84)	2.08 (1.01, 4.28)	5.42 (2.65, 11.09)
	HRS	Strategy 1	2.28 (1.18, 4.40)	3.32 (1.72, 6.41)	5.38 (2.66, 10.89)
	ELSA	Strategy 2	1.68 (0.74, 3.81)	3.54 (1.60, 7.83)	6.24 (2.71, 14.37)
	HRS	Strategy 2	2.14 (1.12, 4.09)	2.52 (1.31, 4.85)	4.46 (2.21, 9.00)

Ref, reference group. For Strategy 1 (BMI + ln(CRP)): Ref = Normal Weight-Low Inflammation, Group 2 = Overweight-Low Inflammation, Group 3 = Obese-Moderate Inflammation, Group 4 = Severe Obese-High Inflammation. For Strategy 2 (WHtR + ln(CRP)): Ref = Low WHtR-Low Inflammation, Group 2 = Moderate WHtR-Low Inflammation, Group 3 = High WHtR-Moderate Inflammation, Group 4 = Very High WHtR-High Inflammation. All comparisons use Model 3 (fully adjusted). HR, hazard ratio; CI, confidence interval; DM, diabetes mellitus; IPTW, inverse probability of treatment weighting.

### Mediation through HbA1c

Mediation analysis was performed to evaluate whether mid-wave HbA1c statistically mediated the trajectory–diabetes association. Higher-risk trajectory groups were associated with elevated HbA1c (a-path), and HbA1c was strongly associated with incident diabetes (b-path HR range: 2.72–2.96, all *p* < 0.001). For the lowest-risk comparison groups, the a-path coefficients were borderline significant in ELSA (*p* = 0.081 and 0.083) but reached significance in HRS (*p* = 0.010 and 0.011), consistent with the non-significant total effects observed in ELSA Cox regression analyses.

To avoid potential inflation arising from the overlap between the mediator (mid-wave HbA1c) and the HbA1c-based component of the diabetes definition, the primary mediation analysis used a conservative outcome definition (physician diagnosis plus glucose-lowering medication only, excluding the HbA1c diagnostic criterion). Under this conservative definition with survival-based mediation, the proportion mediated increased progressively with risk tier: from 13.7 to 24.4% in ELSA and from 16.1 to 27.3% in HRS under the general adiposity strategy, and from 15.0 to 26.9% in ELSA and from 15.2 to 27.5% in HRS under the central adiposity strategy (Table 3). When logistic mediation was applied to the same conservative definition, the proportions were modestly higher but consistent in pattern (Table 3).

**Table 3:** Proportion mediated by mid-wave HbA1c: comparison across outcome definitions and mediation methods.

Cohort	Strategy	Trajectory group	Survival mediation (conservative definition)^a^	Logistic mediation (conservative definition)^a^	Logistic mediation (inclusive definition)^b^
ELSA	BMI + ln(CRP)	Overweight-Low Inflammation	13.7% (6.8, 21.9%)	15.2% (8.3, 23.5%)	25.7% (8.2, 49.8%)
ELSA	BMI + ln(CRP)	Obese-Moderate Inflammation	16.5% (8.2, 26.3%)	18.5% (10.2, 28.7%)	29.1% (16.2, 43.8%)
ELSA	BMI + ln(CRP)	Severe Obese-High Inflammation	24.4% (12.2, 39.1%)	27.0% (14.8, 41.8%)	40.4% (26.8, 56.2%)
HRS	BMI + ln(CRP)	Overweight-Low Inflammation	16.1% (8.0, 25.7%)	17.9% (9.8, 27.7%)	29.2% (11.8, 51.2%)
HRS	BMI + ln(CRP)	Obese-Moderate Inflammation	21.6% (10.8, 34.6%)	24.4% (13.4, 37.9%)	37.5% (22.4, 54.8%)
HRS	BMI + ln(CRP)	Severe Obese-High Inflammation	27.3% (13.6, 43.6%)	30.2% (16.6, 46.9%)	43.7% (28.6, 61.8%)
ELSA	WHtR + ln(CRP)	Moderate WHtR-Low Inflammation	15.0% (7.5, 23.9%)	16.5% (9.1, 25.6%)	28.8% (7.8, 54.8%)
ELSA	WHtR + ln(CRP)	High WHtR-Moderate Inflammation	18.9% (9.5, 30.3%)	21.4% (11.8, 33.2%)	34.5% (18.8, 52.4%)
ELSA	WHtR + ln(CRP)	Very High WHtR-High Inflammation	26.9% (13.4, 43.0%)	29.7% (16.4, 46.1%)	44.8% (28.4, 63.8%)
HRS	WHtR + ln(CRP)	Moderate WHtR-Low Inflammation	15.2% (7.6, 24.3%)	16.8% (9.3, 26.1%)	28.2% (10.8, 49.8%)
HRS	WHtR + ln(CRP)	High WHtR-Moderate Inflammation	20.4% (10.2, 32.6%)	22.9% (12.6, 35.6%)	36.0% (19.8, 54.8%)
HRS	WHtR + ln(CRP)	Very High WHtR-High Inflammation	27.5% (13.8, 44.0%)	30.4% (16.7, 47.0%)	44.8% (27.8, 64.8%)

Reference groups: Normal weight–low inflammation (BMI strategy); low WHtR–low inflammation (WHtR strategy). Values are proportion mediated (95% CI).

a Conservative definition: incident diabetes defined as physician diagnosis or diabetes medication use only, excluding the HbA1c criterion.

b Inclusive definition: incident diabetes defined as physician diagnosis, diabetes medication use, or HbA1c ≥ 6.5%. Estimates under the inclusive definition represent an upper bound due to the mechanistic overlap between the mediator (mid-wave HbA1c) and the outcome definition.

**Figure 5:**
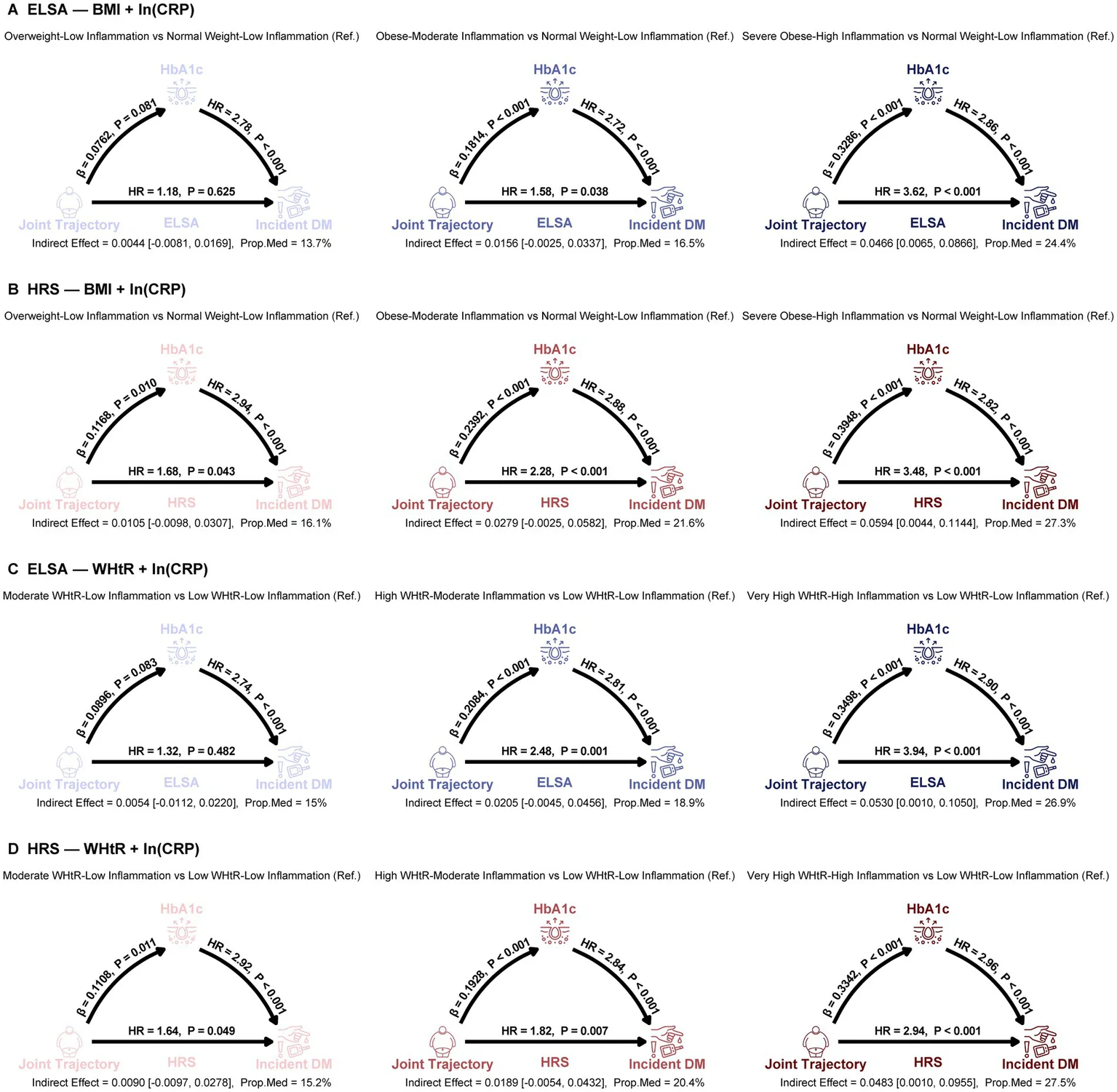
Mediation path diagrams illustrating the role of mid-wave HbA1c in mediating the association between joint trajectory group membership and incident diabetes. Rows **(A)** and **(B)** correspond to ELSA and HRS under Strategy 1 (BMI + ln[CRP]); rows **(C)** and **(D)** correspond to ELSA and HRS under Strategy 2 (WHtR + ln[CRP]). Each diagram displays the a-path (trajectory → HbA1c, linear regression coefficient), b-path (HbA1c → diabetes, hazard ratio from Cox regression), and c′-path (direct effect, hazard ratio). The indirect effect (average mediation effect, ACME) with 95% confidence intervals and the proportion mediated are shown. The primary mediation analysis used a conservative diabetes definition (physician diagnosis plus glucose-lowering medication, excluding the HbA1c diagnostic criterion) with survival-based mediation to avoid inflation from mediator–outcome definitional overlap. Estimates were obtained from quasi-Bayesian Monte Carlo simulation with 1,000 iterations. HbA1c, glycated hemoglobin; DM, diabetes mellitus; HR, hazard ratio.

As a sensitivity analysis representing an upper-bound estimate, mediation was also examined using the original inclusive diabetes definition (incorporating the HbA1c ≥ 6.5% criterion). The proportion mediated was expectedly higher, ranging from 25.7 to 44.8% across cohorts and strategies (Table 3), consistent with the mechanistic overlap between the mediator and the outcome definition (Figure 5).

To assess the robustness of mediation estimates to potential violation of the sequential ignorability assumption, sensitivity analyses were performed using the *ρ* parameter. The crossover ρ values at which the ACME reached zero ranged from 0.30 to 0.42 across all trajectory comparisons, indicating moderate robustness to unmeasured confounding, although residual mediator–outcome confounding at this magnitude remains plausible given the absence of diet and physical activity as covariates (Supplementary Table 4; Supplementary Figure 1B).

### Incremental discriminative value of trajectory classification

**Figure 6:**
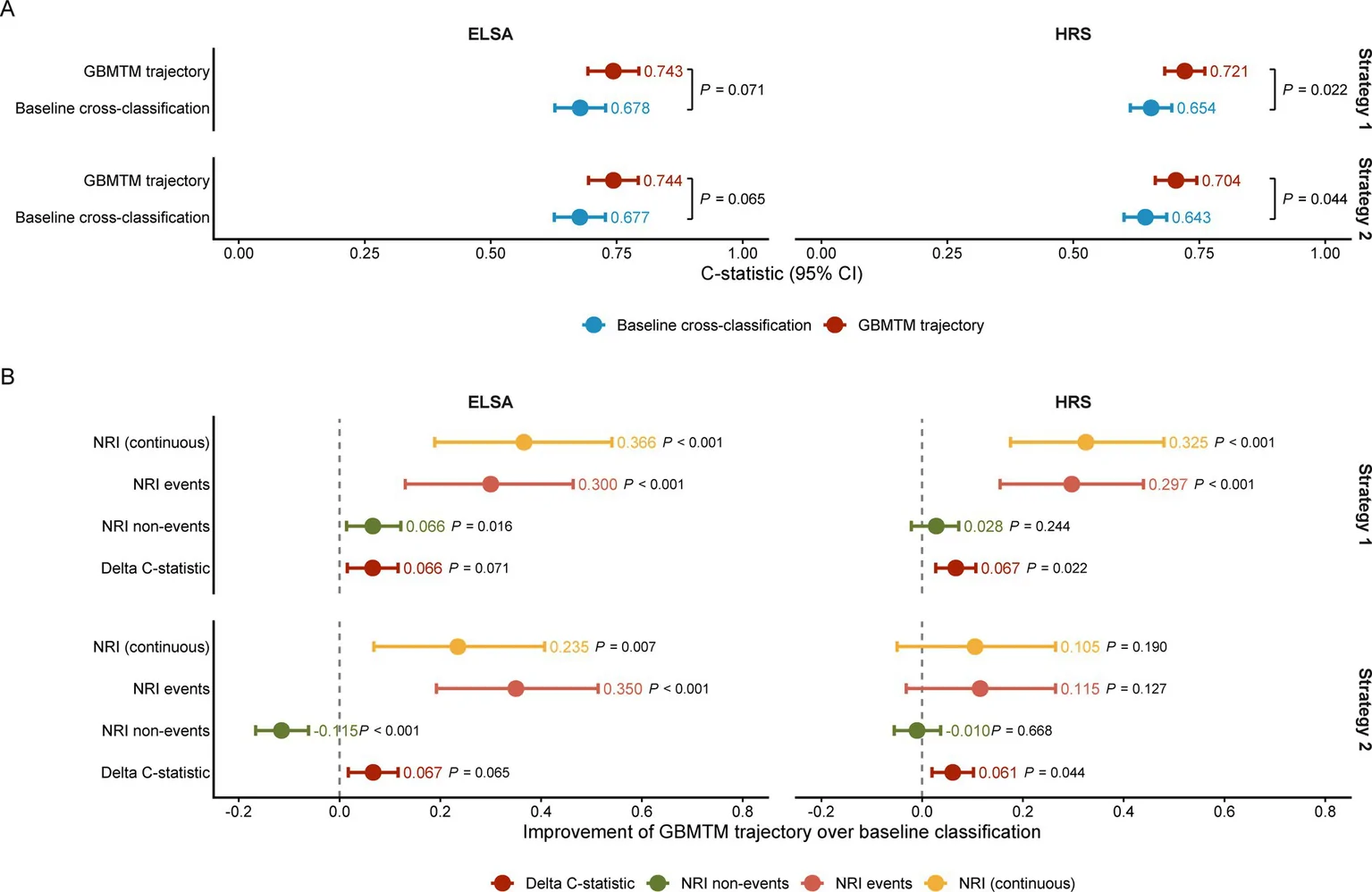
Comparison of discriminative performance between GBMTM joint trajectory classification and baseline adiposity–inflammation cross-classification for incident diabetes. **(A)** Displays C-statistics with 95% confidence intervals from Cox proportional hazards models for baseline cross-classification and GBMTM trajectory models, stratified by cohort and strategy. **(B)** Displays the Delta C-statistic and continuous net reclassification improvement (NRI) with 95% confidence intervals, quantifying the incremental discriminative value of trajectory modeling over baseline classification. The dashed vertical line indicates no improvement.

To evaluate whether GBMTM trajectory classification provided incremental discriminative value beyond baseline adiposity–inflammation cross-classification, C-statistics, information criteria, and continuous net reclassification improvement (NRI) were compared. GBMTM trajectory models yielded consistently higher C-statistics than baseline cross-classification models across both cohorts and strategies. Under the general adiposity strategy, the C-statistic increased from 0.678 to 0.743 in ELSA (ΔC = 0.066, *p* = 0.071) and from 0.654 to 0.721 in HRS (ΔC = 0.067, *p* = 0.022), with concordant improvements under the central adiposity strategy (ELSA: 0.677 to 0.744; HRS: 0.643 to 0.704) (Figure 6; Supplementary Table 5). Trajectory models also demonstrated lower AIC and BIC values in all comparisons, indicating better model fit. Continuous NRI further supported the added discriminative value of trajectory classification, with significant overall NRI observed in ELSA under both strategies (Strategy 1: NRI 0.366, *p* < 0.001; Strategy 2: NRI 0.235, *p* = 0.007) and in HRS under the general adiposity strategy (NRI 0.325, *p* < 0.001), primarily driven by improved reclassification of event cases (Supplementary Table 6). Under HRS Strategy 2, the NRI was positive but did not reach statistical significance (NRI 0.105, *p* = 0.190). Notably, the improvement in C-statistic was modest (ΔC ≈ 0.061–0.067) and reached statistical significance only in HRS, and the NRI was primarily driven by improved reclassification of event cases rather than non-event cases, suggesting that the incremental discriminative value of complex trajectory modeling over single-time-point assessment may be limited.

## Discussion

### Summary of principal findings

This bi-cohort prospective study identified four distinct joint trajectories of adiposity and systemic inflammation using GBMTM in both ELSA and HRS, with concordant trajectory structures emerging independently in each cohort. Under both Strategy 1 (BMI + ln[CRP]) and Strategy 2 (WHtR + ln[CRP]), a clear graded association was observed between escalating adiposity-inflammation trajectory groups and incident diabetes risk, with hazard ratios reaching approximately 6-fold in the highest-risk groups after full covariate adjustment. These associations remained robust across cohorts and were stable across progressive adjustment models. Random-effects meta-analysis confirmed consistent effect directions, although given *k* = 2, heterogeneity statistics should be interpreted with caution. The Overweight–Low Inflammation group in Strategy 1 and the Moderate WHtR–Low Inflammation group in Strategy 2 reached significance in HRS but not in ELSA, likely reflecting differences in sample size and statistical power. Mediation analysis revealed that mid-wave HbA1c statistically mediated 13.7–27.5% of the total effect under the conservative outcome definition excluding the HbA1c criterion. The original inclusive definition yielded higher estimates (25.7–44.8%), but these represent an upper bound given partial overlap between the mediator and outcome ascertainment, with the proportion mediated increasing progressively with trajectory risk tier. Subgroup and sensitivity analyses confirmed the consistency of findings across demographic and clinical strata and under multiple alternative analytic assumptions. Sensitivity analyses using a revised diabetes definition excluding HbA1c and employing survival-based mediation yielded attenuated but significant mediation proportions, and sequential ignorability sensitivity analysis indicated crossover *ρ* values of 0.30–0.42, suggesting moderate robustness to unmeasured confounding, although given the absence of diet and physical activity as covariates, residual mediator–outcome confounding at this magnitude remains plausible. Furthermore, GBMTM trajectory classification yielded modestly higher C-statistics over baseline cross-classification (statistically significant in HRS but not in ELSA), with positive continuous NRI primarily driven by improved reclassification of event cases.

### Comparison with prior literature on adiposity trajectories and diabetes risk

Our findings extend prior work on BMI trajectories and diabetes risk. A systematic review of 14 cohort studies using group-based trajectory modeling concluded that rapidly increasing and stable high-level BMI trajectories conferred the strongest diabetes risk (3). Using data from the Health and Retirement Study, Luo et al. (5) reported that rapidly increasing BMI trajectories in late middle age were associated with an adjusted risk ratio of 1.50 for incident diabetes compared with stable trajectories. In a large Chinese cohort, Zhao et al. (6) demonstrated that high WC trajectory carried a stronger diabetes association (OR 4.34) than high BMI trajectory (OR 2.85), and that individuals maintaining high WC but low BMI still had elevated risk (OR 3.00). Our results are consistent with these observations and further demonstrate that when adiposity trajectories are jointly modeled with inflammatory trajectories, the magnitude of risk estimates increases substantially, with fully adjusted HRs exceeding 5.0 in the highest-risk groups across both strategies. The broadly comparable effect sizes between Strategy 1 and Strategy 2 at corresponding risk tiers suggest that general and central adiposity operate through similar pathways when coupled with chronic inflammation (11, 12).

### The role of inflammation in the adiposity–diabetes pathway

The incorporation of CRP as a joint trajectory component distinguishes our approach from prior single-indicator trajectory analyses. Yang et al. reported a pooled relative risk of 1.77 for T2D comparing extreme CRP quartiles in a meta-analysis of 125,356 participants (4), while Peper et al. found that CRP mediated 10.0% of the racial disparity in incident diabetes among women in the REGARDS study (18). More recently, the CRP-triglyceride-glucose index has been proposed as a composite biomarker integrating metabolic and inflammatory signals, showing strong associations with T2D incidence (HR 3.60) and cardiometabolic multimorbidity (8, 9). Our finding that inflammation and adiposity jointly shape diabetes risk aligns with the inflammatory trajectory framework proposed by Pellegrini et al., which posits that low-grade inflammation both precedes and perpetuates T2D pathogenesis through immune cell activation, epigenetic remodeling, and trained immunity (19). The stability of our effect estimates across adjustment models further indicates that the combined adiposity-inflammation association is not substantially confounded by sociodemographic, behavioral, or comorbidity factors, consistent with an additive rather than purely confounded relationship (1).

### HbA1c as a mediator of the trajectory–diabetes association

Our mediation analysis provides novel evidence that HbA1c partially mediates the pathway from joint adiposity-inflammation trajectories to incident diabetes. Under the original inclusive definition, the proportion mediated (25.7–44.8%) is considerably larger than the 3.7–9.8% mediation by CRP in the coffee–diabetes association reported by Ochoa-Rosales et al. (7), and exceeds the 10.7% mediation by insulin resistance reported by Ning et al. for the BMI trajectory–diabetes pathway (20). The increasing proportion mediated across higher-risk trajectory groups parallels the observation by Yu et al. that obese individuals exhibit steeper pre-diagnostic HbA1c trajectories than non-obese individuals (10), and is consistent with findings that HbA1c trajectory patterns strongly predict diabetes complications (21, 22). The borderline a-path significance for the lowest-risk comparison groups in ELSA, coupled with their non-significant total effects in Cox regression, indicates internal coherence of the mediation findings and suggests that at lower adiposity-inflammation levels, the glycemic pathway may not be sufficiently activated (23, 24). An important methodological consideration is that mid-wave HbA1c partially overlaps with the HbA1c-based component of the original diabetes outcome definition, which could inflate the proportion mediated. To address this concern, we re-estimated the mediation using a revised definition based solely on physician diagnosis and glucose-lowering medication. The proportion mediated was attenuated—for example, from 40.4 to 27.0% in ELSA and from 43.7 to 30.2% in HRS for the highest-risk group under Strategy 1 with logistic mediation—and remained statistically significant for the higher-risk trajectory groups, although the lowest-risk comparison groups did not reach significance under the revised definition in either cohort. Survival-based mediation further reduced the estimates (to 24.4 and 27.3%, respectively), suggesting that although the original analysis likely overestimated the mediating contribution, HbA1c retains a meaningful role as a mediator of the adiposity-inflammation-diabetes pathway under conservative outcome definitions, particularly for the higher-risk trajectory groups. Importantly, the conservative estimates (13.7–27.5%) should be considered the primary mediation findings, as they eliminate the mechanical inflation introduced by the overlap between the mediator and the HbA1c-based outcome criterion.

### Strengths and limitations

This study has several strengths. The use of two independent, nationally representative cohorts with parallel GBMTM modeling—in which trajectory groups were identified independently in each cohort rather than imposing a model derived from one onto the other—enhances reproducibility and avoids overfitting to a single population. The dual-strategy design enables direct comparison of general versus central adiposity indicators within a unified analytic framework. Progressive covariate adjustment, verification of the proportional hazards assumption, and comprehensive sensitivity analyses—including competing risk models, complete case analysis, IPTW, and alternative CRP specifications—strengthen internal validity. However, several limitations should be acknowledged. First and most importantly, the analytic sample retained only approximately 19% of ELSA and 11% of HRS participants initially identified, primarily due to biomarker missingness. In HRS, CRP is derived from a biomarker subsample, meaning that the analytic cohort represents a non-random subset. Comparison of included and excluded participants revealed that included individuals were more likely to be married, non-smoking, and free of cardiovascular comorbidities, although incident diabetes rates were comparable between groups (Supplementary Table 1). This selection likely favored healthier participants, and the resulting estimates may bias the adiposity-inflammation-diabetes association, most plausibly toward the null, although the direction cannot be definitively established given evidence of effect modification by comorbidity status in subgroup analyses. The findings are therefore applicable primarily to relatively healthy older adults with complete biomarker data and may not generalize to the broader aging population. CRP was measured at each wave rather than as high-sensitivity CRP in all instances, potentially introducing measurement variability. Specifically, ELSA used immunoturbidimetric assays on venous blood, whereas HRS used latex-enhanced immunoturbidimetric assays on dried blood spots, and standard CRP rather than high-sensitivity CRP was used at certain waves. Values below the detection limit were set to half the limit prior to log transformation. These inter-cohort and inter-wave assay differences may contribute to the observed decline in mean ln(CRP) in HRS across waves (from 0.66 to 0.25), which could reflect assay variability or differential dropout rather than a true biological trend. Diabetes ascertainment relied partly on self-report, which may underestimate incidence. Neither diet nor physical activity was included as a covariate, which represents a notable residual confounding gap given the study’s framing around nutritional excess and inflammatory burden. An important design limitation is that trajectory group membership was defined using data spanning the entire observation window, including the mid-wave at which the mediator (HbA1c) was assessed. The trajectory groups are therefore identified retrospectively, and group assignment may incorporate post-baseline trends partly driven by preclinical disease, introducing a risk of protopathic bias. Consequently, the observed associations should be interpreted as discriminative associations between retrospectively identified trajectory patterns and diabetes incidence, rather than as prospective predictions. Although ELSA and HRS collect limited dietary data, the absence of these variables means that part of the observed trajectory-diabetes association may be attributable to unmeasured lifestyle factors that influence both adiposity-inflammation trajectories and diabetes risk. The original mediation analysis used logistic regression for the outcome model, which does not account for censoring and time-to-event. To address this limitation, the primary mediation analysis employed survival-based mediation using the revised diabetes definition; the resulting estimates were modestly attenuated but qualitatively consistent (Supplementary Table 4), suggesting that the choice of outcome model did not materially alter the conclusions. Additionally, the predominantly White European and American populations in ELSA and HRS limit generalizability to other ethnic groups, particularly given ethnicity-specific BMI thresholds for diabetes risk (25). Finally, pooling cohort-specific estimates using DerSimonian-Laird random-effects meta-analysis with only two studies (*k* = 2) yields unreliable estimates of between-study variance (τ^2^) and heterogeneity (*I*^2^). Cohort-specific hazard ratios are therefore presented alongside the pooled estimates in Figure 4 to allow readers to evaluate consistency directly, and the concordance between ELSA and HRS estimates should be interpreted as parallel replication rather than formal meta-analytic synthesis.

### Clinical implications and future directions

Our findings carry several implications for clinical practice. The identification of distinct adiposity-inflammation trajectory phenotypes supports a move beyond single-time-point BMI assessment toward longitudinal monitoring of both adiposity and inflammatory markers for diabetes risk stratification (15, 26). The partial mediating role of HbA1c suggests that early glycemic surveillance among individuals with high adiposity-inflammation burden may facilitate timely intervention before diabetes onset (27, 28). The concordance between BMI-based and WHtR-based strategies indicates that either measure, when combined with CRP, provides robust risk discrimination, offering flexibility in clinical settings where waist circumference measurement may be more practical. Future studies should examine whether pharmacological or lifestyle interventions targeting inflammation—such as GLP-1 receptor agonists or anti-inflammatory therapies—can modify the inflammatory trajectory and thereby reduce diabetes incidence in high-risk subgroups (2, 19). Replication in ethnically diverse populations and integration of additional inflammatory mediators beyond CRP would further strengthen the evidence base (14, 29).

## Conclusion

In this bi-cohort study of 3,733 middle-aged and older adults from England and the United States, joint trajectories of adiposity and systemic inflammation were independently and consistently associated with incident type 2 diabetes in a graded manner, with the highest-risk trajectory groups exhibiting approximately six-fold elevated risk after full adjustment. These associations were comparable between general adiposity (BMI) and central adiposity (WHtR) strategies and were consistently observed across two independent populations. Mid-wave HbA1c mediated 13.7–27.5% of the trajectory–diabetes association under the conservative outcome definition excluding HbA1c (25.7–44.8% under the original definition, representing an upper bound), with mediation significant for the higher-risk trajectory groups, supporting glycemic deterioration as a mediating pathway. These findings support the integration of longitudinal adiposity and inflammatory biomarker monitoring for diabetes risk stratification, although the modest incremental discriminative gain over single-time-point assessment should be considered when weighing the additional data requirements. Glycemic deterioration represents a partial mediating pathway linking combined adiposity-inflammation burden to diabetes development.

## Data Availability

Publicly available datasets were analyzed in this study. ELSA data can be accessed through the UK Data Service (https://ukdataservice.ac.uk/). HRS data can be accessed through the Health and Retirement Study website (https://hrs.isr.umich.edu/).

## Supplementary material

SUPPLEMENTARY FIGURE 1 Sensitivity analyses of the mediation of mid-wave HbA1c in the association between joint trajectory group membership and incident diabetes. Panel A displays the proportion mediated with 95% confidence intervals under three analytic approaches: original diabetes definition with logistic mediation **(A)**, revised definition excluding HbA1c criterion with logistic mediation **(B)**, and revised definition with survival-based mediation **(C)**, stratified by cohort and strategy. Panels B–E display the average causal mediation effect (ACME) as a function of the sensitivity parameter *ρ*, which represents the correlation between residuals of the mediator and outcome models under violation of the sequential ignorability assumption. Cross markers indicate the crossover *ρ* value at which ACME equals zero. Analyses in Panels B–E were conducted under the revised diabetes definition. **(B)** ELSA — BMI + ln(CRP); **(C)** HRS — BMI + ln(CRP); **(D)** ELSA — WHtR + ln(CRP); **(E)** HRS — WHtR + ln(CRP).
